# Positive relationship between odor identification and affective responses of negatively valenced odors

**DOI:** 10.3389/fpsyg.2015.00607

**Published:** 2015-05-11

**Authors:** Lenka Martinec Nováková, Dagmar Plotěná, S. Craig Roberts, Jan Havlíček

**Affiliations:** ^1^Department of Anthropology, Faculty of Humanities, Charles UniversityPrague, Czech Republic; ^2^Department of Zoology, Faculty of Science, Charles UniversityPrague, Czech Republic; ^3^School of Natural Sciences, University of StirlingStirling, UK

**Keywords:** food, smell, children, pleasantness, olfactory abilities, hedonic evaluation, odor preferences

## Abstract

Hedonic ratings of odors and olfactory preferences are influenced by a number of modulating factors, such as prior experience and knowledge about an odor’s identity. The present study addresses the relationship between knowledge about an odor’s identity due to prior experience, assessed by means of a test of cued odor identification, and odor pleasantness ratings in children who exhibit ongoing olfactory learning. Ninety-one children aged 8–11 years rated the pleasantness of odors in the Sniffin’ Sticks test and, subsequently, took the odor identification test. A positive association between odor identification and pleasantness was found for two unpleasant food odors (garlic and fish): higher pleasantness ratings were exhibited by those participants who correctly identified these odors compared to those who failed to correctly identify them. However, we did not find a similar effect for any of the more pleasant odors. The results of this study suggest that pleasantness ratings of some odors may be modulated by the knowledge of their identity due to prior experience and that this relationship might be more evident in unpleasant odors.

## Introduction

Preferences in adults can be described as “relatively stable evaluative judgments in the sense of liking or disliking a stimulus, or preferring it or not over other objects or stimuli” (Scherer, 2005). More specifically, olfactory preferences have been shown to have a profound impact on human psychology and behavior in varied aspects of life such as ingestion, environmental hazards, and social interactions (Stevenson, 2010). It is, therefore, important to understand the formation of these affective responses to odors and the effects of factors that may modulate them across the lifespan (for review see Rouby et al., 2009). The widely accepted view is that humans are not born with any fixed set of olfactory likes or dislikes and that affective responses toward odors are to a great extent shaped by evaluative conditioning (Herz, 2006), starting as early as in the pre- and perinatal period (Marlier et al., 1998) and continuing in the context of everyday individual experience with odors within one’s culture. Thus, certain odors are encountered more frequently than others in specific contexts and, as a result, are attributed with a locally specific meaning and hedonic value which people outside this cultural setting may not share. For example, in a cross-cultural study by Ayabe-Kanamura et al. (1998), significant differences in odor naming performance (also referred to as “free identification”) and ratings of pleasantness, edibility, and intensity between German and Japanese women were noted for many culture-specific odors, suggesting the crucial effect of odor familiarity on olfactory perception and ratings of pleasantness in particular.

Experience with odors constitutes a major factor modulating olfactory perception. It is thus frequently found that ratings of familiarity of a given odor are positively associated with ratings of pleasantness (Royet et al., 1999; Sulmont et al., 2002), although this finding does not invariably reach statistical significance (Savic and Berglund, 2000; Bensafi et al., 2002) or is not consistent across studies (Distel et al., 1999). Delplanque et al. (2008) have demonstrated that the strength of the association differs as a function of average odor pleasantness, with odors rated as pleasant exhibiting positive correlations with ratings of familiarity. However, no similar association was found for the unpleasant odors. This finding has recently been corroborated cross-culturally by Ferdenzi et al. (2013), who reported that the relationship between odor knowledge and affective response was generally asymmetrical and significant only for the pleasant odors, whereas the unpleasant ones seemed more resistant to cognitive modulation. In a similar vein, Konstantinidis et al. (2006) have demonstrated that identification of unpleasant odors (but not pleasant ones) was relatively independent of age. Finally, using the test of odor identification as a proxy for odor experience, Knaapila et al. (2007) have shown that some odors, which varied significantly in terms of mean pleasantness, were evaluated as more pleasant when correctly identified than when not. Overall, unpleasant odors tend to be less susceptible to cognitive and contextual effects.

The major body of evidence comes from studies with adult participants, who have already acquired substantial odor semantic knowledge, but this may be somewhat different in children. Indeed, although olfactory perception is extensively shaped by experience, affective responses to some biologically relevant odors appear to be independent of previous experience (Soussignan et al., 1997). As children have lower levels of odor semantic knowledge, their hedonic perception could be more influenced by the physicochemical properties of odors. Several previous studies have shown that odorant structure can predict hedonic perception (e.g., Khan et al., 2007; Mandairon et al., 2009) and this may occur in a manner that is dependent on the age of the participants. Specifically, Poncelet et al. (2010) measured hedonic response to odors in different age groups and reported a pronounced role of physicochemical properties in processing of odor hedonics in (prepubertal) children and elderly people, who, respectively, exhibit either a low level of, or a weak access to, odor semantic knowledge. This was in contrast to teenagers and young adults, who are characterized by higher levels of semantic odor representation. Among the physicochemical properties of odorants that can make an odor *a priori* unpleasant are those related to trigeminal stimulation (pungency; Herz, 2006), which triggers neurological protective reactions that help avert the organism from potentially harmful materials (for a review see Doty and Cometto-Muñiz, 2003).

The aim of the present study was to explore the relationship between knowledge of an odor’s identity (assessed by means of performance on a cued identification task) and pleasantness ratings in a cohort of prepubertal children, who have less experience with odors than adults and in whom the process of odor knowledge acquisition is evident from their increase in odor identification scores with age (Ferdenzi et al., 2008). Although inclusion of preschool children would have been particularly informative, recruitment of slightly older children helped prevent several methodological issues related to limitations on young children’s attention span and motivation. We hypothesized that an odor would be rated as more pleasant when identified correctly, aiming to assess whether the previously reported positive relationship between odor pleasantness and olfactory knowledge could be generalized to an age group that clearly exhibits ongoing olfactory learning. In so doing, we used a cued odor identification task on which Czech children perform well (Dudova et al., 2011; Hrdlicka et al., 2011) and for which individual odor identification rates as well as pleasantness ratings in the adult European population across the lifespan are well-established (e.g., Konstantinidis et al., 2006).

## Materials and Methods

### Participants

The participants were 91 children of Czech origin (36 boys, mean age 9.31 ± 0.73, range 8–11 years), who were third (*N* = 44; 15 boys) and fourth graders (*N* = 47; 21 boys) from two mixed-sex general education elementary schools. There was no significant difference in the proportion of boys and girls across grades in the sample, χ^2^(1) = 1.12, *p* = 0.29, and they did not differ in terms of mean age or age distribution, boys = 9.44 ± 0.82 and girls = 9.24 ± 0.67 years, respectively, *t*(59.14) = 1.22, *p* = 0.23. Two cases (boys) were not included in the analysis because the absolute distance of their ratings from the median exceeded the cut-off based on the median absolute deviation (Wilcox, 2010) for 8 out of 16 odors, and, at the same time, their ratings represented extremes in two out of the total of four plots in which outliers and extremes were visually detected.

All procedures followed were in accordance with the ethical standards of the responsible committee on human experimentation (institutional and national) and with the Helsinki Declaration of 1975, as revised in 2008 (5). The study was approved by the IRB of the Charles University (Approval Number 2008/4). The children’s parents provided written informed consent.

### Olfactory Measures

Olfactory assessment included ratings of odor pleasantness and an odor identification test. We used the 16-item Sniffin’ Sticks odor identification test, a psychophysical test of orthonasal chemosensory performance based on pen-like odor dispensing devices. The Sniffin’ Sticks test has been widely used by clinicians and researchers across Europe to test olfactory abilities in adults (Hummel et al., 2007b) and children (Ferdenzi et al., 2008; Renner et al., 2009; Dudova et al., 2011; Hrdlicka et al., 2011). The identification test consists of odorants familiar to the general European population, such as orange, rose, garlic, and fish (full list in **Table 1**). Cued identification is employed, in which participants select the name of the target odor from a candidate list of four. The resulting score is the sum of correct answers, which can vary between 0 and 16, with 4 as a chance score (Hummel et al., 1997). The same set of odorants was used to obtain category ratings of odor pleasantness, which copied the system of grading used in Czech schools (1 being the best grade achievable and 5 being the failing grade) to facilitate scale comprehension by this age group (1 = very pleasant odor, 5 = very unpleasant odor). The scores were subsequently recoded to 1 = very unpleasant, 5 = very pleasant.

**Table 1:** Percentages of correct identifications and mean pleasantness for individual items of the Sniffin‘ Sticks identification test (*N* = 89).

Item	Percent identified	95% Confidence intervals	Mean ± SD Pleasantness		
			Overall	Correctly identified	Not identified
Orange	40.4%	30.9, 50.8	4.26 ± 1.05	4.22 ± 1.05	4.28 ± 1.062
Leather	47.2%	37.2, 57.5	2.90 ± 1.31	2.76 ± 1.34	3.02 ± 1.29
Cinnamon	78.7%	69, 85.9	3.94 ± 1.14	3.86 ± 1.17	4.263 ± 0.99
Mint	86.5%	77.9, 92.1	4.08 ± 1.07	4.03 ± 1.09	4.42 ± 0.90
Banana	89.9%	81.9, 94.6	4.16 ± 1.09	4.18 ± 1.08	4.00 ± 1.22
Lemon	32.6%	23.7, 42.9	3.53 ± 1.27	3.76 ± 1.09	3.42 ± 1.34
Liquorice	60.7%	50.3, 70.2	3.49 ± 1.28	3.48 ± 1.28	3.51 ± 1.29
Turpentine	31.5%	22.8, 41.7	2.51 ± 1.11	2.50 ± 1.26	2.51 ± 1.04
Garlic	75.3%	65.4, 83.1	2.08 ± 1.28	2.21 ± 1.31	1.68 ± 1.13
Coffee	77.5%	67.8, 85	1.99 ± 1.17	2.07 ± 1.20	1.70 ± 1.03
Apple	10.1%	5.4, 18.1	3.90 ± 1.18	4.22 ± 0.83	3.86 ± 1.21
Clove	73.0%	63, 81.2	2.07 ± 1.15	2.05 ± 1.18	2.13 ± 1.08
Pineapple	57.3%	46.9, 67.1	3.61 ± 1.35	3.61 ± 1.40	3.61 ± 1.31
Rose	55.1%	44.7, 65	4.08 ± 1.15	4.06 ± 1.21	4.10 ± 1.08
Anise	38.2%	28.8, 48.6	3.16 ± 1.22	2.85 ± 1.13	3.35 ± 1.25
Fish	69.7%	59.5, 78.2	1.66 ± 1.00	1.74 ± 0.94	1.48 ± 1.12

Note that pleasantness ratings have been recoded (1 = least pleasant, 5 = most pleasant).

### Procedure

The children participated in individual testing sessions, which were scheduled for morning during school time, to avoid possible diurnal fluctuations in olfactory abilities. The testing took place in a quiet, ventilated room without strong ambient odors. The stimuli were presented in the order recommended by Hummel et al. (1997) for the standard procedure. The presentation of each stimulus took approximately 5 s. Subsequent stimuli were presented immediately after the participant selected a verbal label/pleasantness rating for the previous stimulus. Since a verbal label may affect hedonic perception (e.g., Herz, 2003), ratings of pleasantness were obtained first for all odors, followed by the task of odor identification. Subsequently, the participants were interviewed about their odor awareness using the COBEL questionnaire (Ferdenzi et al., 2008). The part on odor awareness has been published elsewhere (Saxton et al., 2014) and is not further reported here.

### Statistical Analysis

All analyses were carried out with IBM SPSS 22.0. Normality of the raw data was checked for each odor separately. Firstly, we produced skewness and kurtosis values and their respective SEs, from which *z*-scores were computed and compared to the value of 1.96, as suggested by Field (2005). Secondly, we visually examined individual histograms of all relevant variables. Finally, we ran the Shapiro–Wilk’s *W* test for each variable. Since the results of the Shapiro–Wilk’s test, visual examination of the respective histograms, and skewness *z*-scores all indicated that the pleasantness ratings of each individual odor departed significantly from normality, non-parametric tests were employed where possible.

#### Descriptive Statistics

Based on the method proposed by Bonett and Price (2002), we computed 95% confidence intervals (95% CI) for median pleasantness of each odor. Confidence intervals for the proportions of correct identifications were computed following the method recommended by Newcombe and Altman (2000).

**FIGURE 1:**
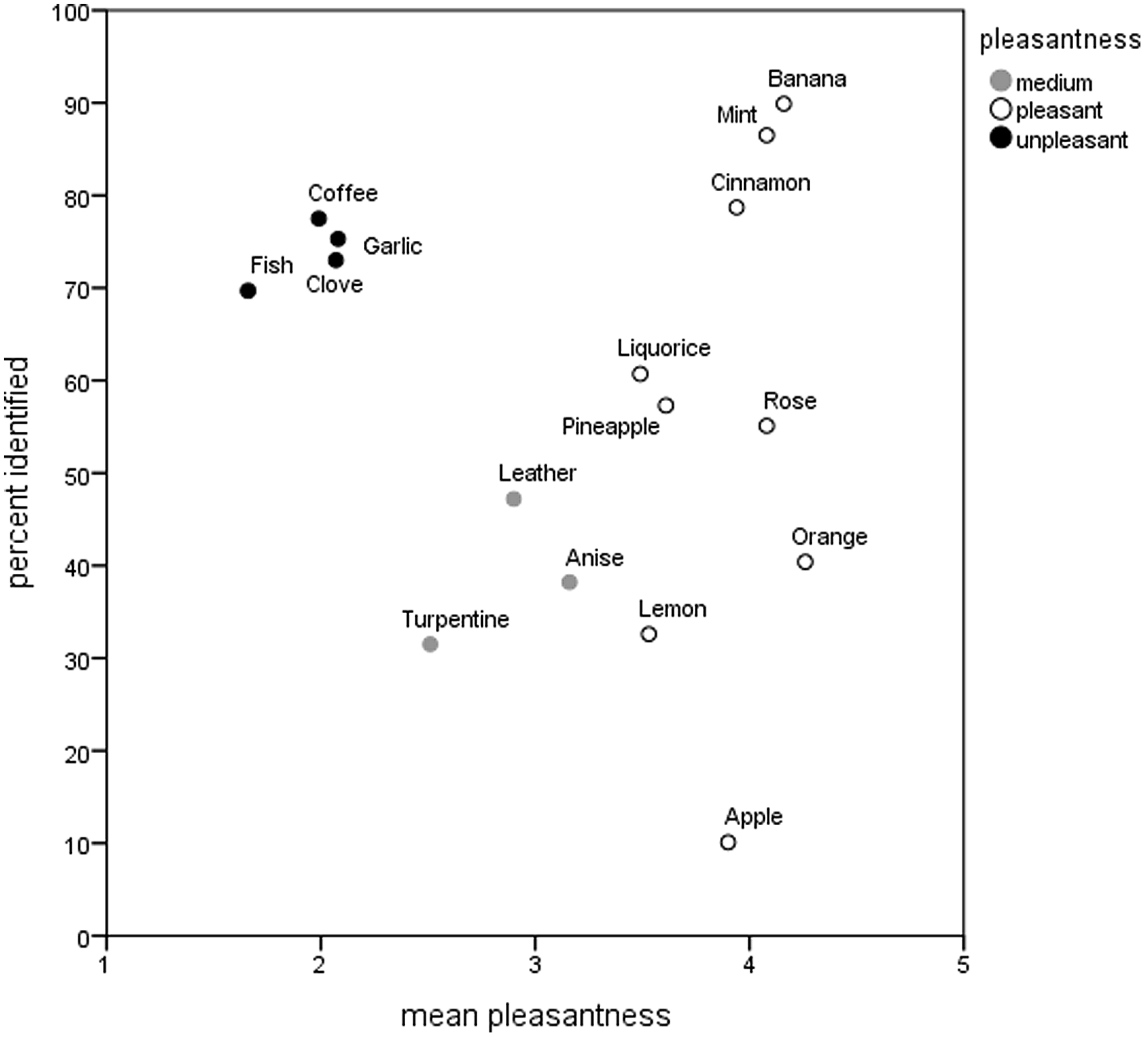
**Scatter plot of mean pleasantness ratings and percentages of correct identifications for the 16 odors of the Sniffin’ Sticks odor identification test.** The pleasant and unpleasant subsets are given in white and black, respectively, and the medium pleasant odors are given in gray.

To test whether association between odor identification and pleasantness ratings is limited to unpleasant odors, we aimed to classify the odors on the basis of their median pleasantness. The median pleasantness values for each odor were entered into a two-step cluster analysis, in which we predefined three clusters in the solution and used default settings. Although a Shapiro–Wilk test showed that the assumption of normality was not met, *W* = 0.862, df = 16, *p* = 0.021, the procedure is considered fairly robust to violations of the assumption (IBM SPSS, 2012). Since the final solution may depend on the order of cases, to verify the stability of the solution, several trials with randomly ordered cases were run. The analysis repeatedly yielded a model of good cluster quality (average silhouette of 0.8). The group of pleasant odors included the odors of orange [median pleasantness rating of 5; 95% CI (4.49, 5.51)], apple, banana, cinnamon, lemon, liquorice, mint, pineapple, and rose [all with a median pleasantness rating of 4; 95% CIs (3.49, 4.51)]. The group of unpleasant odors consisted of the odor of fish (median pleasantness rating of 1), clove, coffee, and garlic [each with a median pleasantness rating of 2, 95% CIs (-1.77, 3.39)]. The remaining odors (anise, leather, and turpentine) all received a median pleasantness rating of 3; 95% CIs (2.49, 3.51). The mean pleasantness values for the three groups are depicted in **Figure 1**.

The percentages of correct identifications and mean pleasantness ratings for each of the odors are given in **Table 1**.

#### Correlational Analyses of Odor Identification Scores and Pleasantness Ratings

To test for any overall association between individual children’s performance scores on the odor identification test and their median pleasantness ratings given to the odors, Kendall’s Tau correlations were performed. These analyses were performed on averages per participant of, firstly, all the 16 odors, secondly, the subset of nine pleasant odors (median pleasantness of 4), and thirdly, the subset of four unpleasant odors (median pleasantness of 2).

#### Odor-Specific Analyses: Odor Identification as a Predictor of Odor Pleasantness

Finally, to test whether the sought effect could be limited to certain individual odors, rather than spanning whole odor subsets, we performed odor-specific analyses. First, to determine whether children’s pooled responses could be conceived of as a homogeneous sample, we tested for the effect of sex and age on odor identification performance and pleasantness ratings of the individual odors, respectively. Both of these variables are known to affect odor identification in children (Ferdenzi et al., 2008). To do this, we ran multiple Categorical Regression (CATREG) analyses using the IBM SPSS (2012) Optimal Scaling option. The independent variables of sex and age were treated as nominal and numeric, respectively, and the dependent variables of identification performance and pleasantness rating were scaled as nominal and spline ordinal, respectively. Both the nominal variables were categorized into groups of two, and the numeric and spline ordinal variables by ranking. A random initial configuration was selected, as recommended in cases in which at least one of the predictors has a nominal scaling level. The rest of the options were left to default settings. Subsequently, predictions of individual odor pleasantness with odor identification (a yes/no response) were modeled in the same manner, using identical settings.

## Results

### Correlational Analyses of Odor Identification Scores and Pleasantness Ratings

Correlational analyses revealed no significant association between children’s total identification scores and their mean pleasantness ratings for the complete set of odors, Kendall’s Tau-*b* = -0.07, *p* = 0.36, *N* = 89 (**Figure 1**). That is, children who tended to correctly identify more odors than others did not exhibit any tendency toward higher ratings of pleasantness in general. Nor was there such an association found for the subsets of pleasant, medium, and unpleasant odors analyzed separately, Kendall’s Tau-*b* = 0.01, Kendall’s Tau-*b* = 0.02, *N* = 89, and 0.04, *N* = 89, all *p*s > 0.05, respectively. For exploratory purposes we also fitted a quadratic regression model to the data which however was not significant (*p* > 0.1). Relative frequencies of correct identification and mean pleasantness ratings for the individual odors can be found in **Table 1**.

### Odor-Specific Analyses: Odor Identification as a Predictor of Odor Pleasantness

First, to test whether participant characteristics (sex and age) predicted odor identification and pleasantness ratings, multiple CATREG analyses were run. These showed that identification of the odor of orange was predicted by sex, β = 0.25, *F* = 6.96, *p* < 0.01, and age, β = 0.21, *F* = 5.40, *p* < 0.05, with girls and older children being more likely to correctly identify the odor. Also, sex (but not age) predicted pleasantness ratings of orange, β = 0.23, *F* = 6.24, *p* < 0.05, with girls (mean 4.35 ± 0.91 SD) rating the odor as more pleasant than boys (mean 4.12 ± 1.25 SD). However, both models only explained about 9% of the total variance in identification and pleasantness of orange, *R*^2^ = 0.095, *F*(2,88) = 4.50, *p* < 0.05 and *R*^2^ = 0.093, *F*(2,88) = 4.39, *p* < 0.05. Further, sex (but not age) also predicted pleasantness ratings of the odor of apple, β = 0.24, *F*(1) = 5.76, *p* < 0.05, again with girls (mean 4.09 ± 1.08 SD) giving higher pleasantness ratings to the odor than boys (mean 3.59 ± 1.28 SD). The overall model was significant but only explained 7.3% of the total variance in pleasantness ratings of the odor of apple, *R*^2^ = 0.07, *F*(2,88) = 3.37, *p* < 0.05. Thus, for the odors of orange and apple, sex was included as a predictor in the subsequent analyses. There were no significant sex and age effects on identification or pleasantness ratings of any other odorants.

**FIGURE 2:**
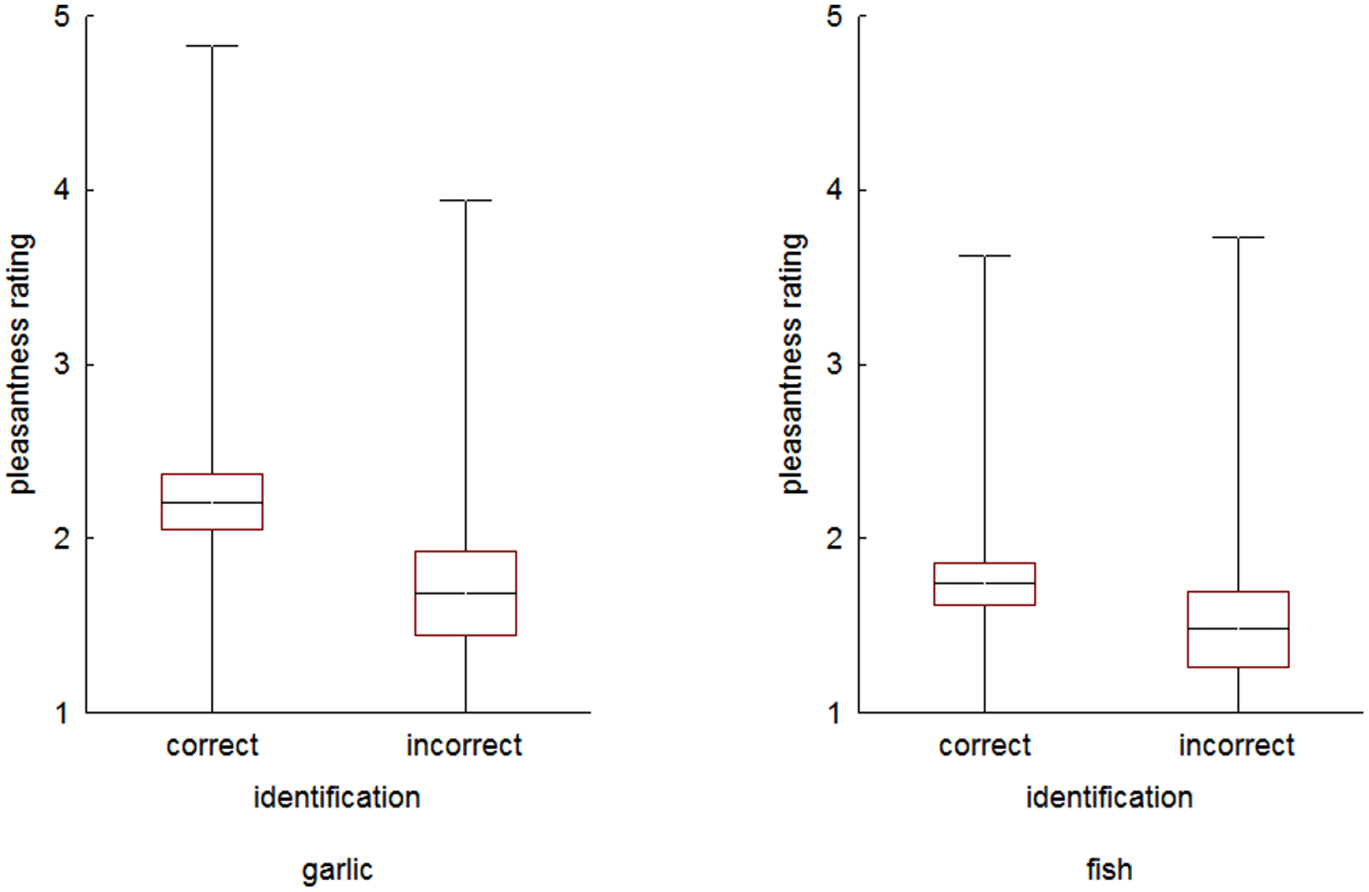
**Ratings of pleasantness in children who correctly identified and those who did not for the odors of garlic and fish.** Middle line denotes mean, boxes ± SEM and error bars ± 2SD. The differences are significant at *p* > 0.05.

Second, and more importantly, identification significantly predicted odor pleasantness in two cases: firstly, in the odor of garlic, β = 0.24, *F* = 7.75, *p* < 0.01; *R*^2^ = 0.06, *F*(1,88) = 5.36, *p* < 0.05, and, secondly, in the odor of fish, β = 0.25, *F* = 6.97, *p* < 0.01; *R*^2^ = 0.06, *F*(1,88) = 5.56, *p* < 0.05. In both cases higher pleasantness ratings were given to these odors by children who correctly identified them (**Figure 2**). No significant relationship between odor identification and pleasantness was found for any of the other tested odors (**Table 2**).

**Table 2:** Categorical regression (CATREG) analysis for predicting odor pleasantness from identification (correct/incorrect) for the individual odors.

	Model			Identification		
	*R*^2^	*F*	*p*	β	*F*	*p*
Orange	0.044	1.970	0.146	0.076	0.771	0.382
Leather	0.030	2.698	0.104	0.173	2.329	0.131
Cinnamon	0.027	2.407	0.124	0.164	3.229	0.076
Mint	0.026	2.295	0.133	0.160	3.569	0.062
Banana	0.013	1.188	0.279	0.116	1.438	0.234
Lemon	0.032	2.843	0.095	0.178	5.244	0.024
Liquorice	0.001	0.044	0.835	0.022	0.088	0.767
Turpentine	0.041	3.755	0.056	0.203	4.524	0.036
**Garlic**	**0.058**	**5.359**	**0.023**	**0.241**	**7.748**	**0.007**
Coffee	0.028	2.541	0.115	0.168	4.637	0.034
Apple	0.014	1.269	0.263	0.120	5.222	0.025
Clove	0.017	1.538	0.218	0.132	9.889	0.002
Pineapple	0.008	0.662	0.418	0.087	1.059	0.306
Rose	0.001	0.107	0.745	0.035	0.202	0.654
Anise	0.050	4.550	0.036	0.223	3.225	0.076
**Fish**	**0.060**	**5.559**	**0.021**	**0.245**	**6.965**	**0.009**

Odors for which both the model and predictor were significant at *p* < 0.05 are given in bold.

## Discussion

The key objective of the present study was to explore the relationship between children’s knowledge of an odor’s identity, assessed with a cued odor identification test, and pleasantness ratings given to these odors. The results show that identification success or failure only predicted odor pleasantness in the two cases of garlic and fish, both of which also happened to fall among the unpleasant odors. The two odors tended to be given higher ratings of pleasantness by children who could identify them correctly than by those who could not.

### The Relation of Odor Identification and Pleasantness

In the study by Knaapila et al. (2007) with adult participants, the odors of cinnamon, lemon, rose, and banana were evaluated as more pleasant, and turpentine as less pleasant, by individuals who had identified them correctly compared with those who had not, suggesting that the association between knowledge of an odor’s identity, assessed with an odor identification test, and odor pleasantness may take different directions for different odors. The positive relationship between odor identification and pleasantness was reported for odors which were on average rated as relatively more pleasant (Knaapila et al., 2007). Similarly, Mennella and Forestell (2008) found in 5 to 8-year-olds higher identification rates in the odors they liked (bubble gum, strawberry, chocolate). However, the direct comparison with this study might be limited due to the differences in odor identification assessment (the former study employed a free odor identification task while in the present study we used a cued identification test). In contrast to both studies, in the present study a positive association was found for two of the four unpleasant odors. To further complicate this issue, Bensafi et al. (2007) showed that a shift in pleasantness ratings in correctly identified odors was limited only to those judged on average as neutral. The apparent discrepancies across the individual studies point to the complexity of the association between odor identification and pleasantness. This might be due to modulating factors which were not controlled for in the previous studies and, as a consequence, the association between odor identification and pleasantness might sometimes be limited to pleasant, neutral, or even unpleasant odors, as in the current study. Such modulating factors may include variation in pleasantness, familiarity, edibility, or pungency of the employed set of odorants. Researchers should address these issues while designing future studies to clarify reasons for these apparent discrepancies.

Furthermore, in our study, the positive relationship did not pertain to all odors rated as rather unpleasant but was limited to garlic and fish, whereas pleasantness ratings of the other two unpleasant odors (coffee and clove), which exhibited similar pleasantness ratings and percentages of correct identifications, were not related to identification success or failure. Consequently, this raises the question of how, besides the variables assessed within the present study, these two odors might differ from those of fish and garlic. One explanation may stem from the fact that the participants were children: unlike garlic and fish, coffee and clove may not be categorized as food odors by children. In the case of coffee, the obvious reason would be that most exposure to this odor in Czech children of this age group is through its presence in the children’s close, everyday environment but not through direct consumption. Indeed, reports of coffee consumption in prepubertal children in various European countries show rather negligible values (Meltzer et al., 2008; Duffey et al., 2012; Ng et al., 2012) and a flavor preference study showed coffee to be amongst the least preferred in this age group, as well as in younger children (Liem et al., 2010). The odor of clove, in adults at least, tends to be associated with experiences at the dentist’s rather than with food. For instance, in a study that assessed autonomic emotional responses to odors, it was found that the clove-smelling odorant eugenol, which is used in dentistry, was given very low pleasantness ratings and elicited autonomic reactions indicative of stress in participants who feared dental procedures (Robin et al., 1998, 1999). However, formation of this association in young children will be comparatively rare. Thus, the odors of coffee and clove may differ from the equally unpleasant odors of garlic and fish in that they may be less relevant to their everyday life. Unpleasant stimuli seem to constitute a unique odor category, e.g., they elicit faster and more accurate reactions since they may signal a potential danger (Boesveldt et al., 2010). It is for just this kind of odor that we would most expect to see changes in perception with increasing familiarity – where initial odor unpleasantness can be modulated by a learned association with food. Alternatively, but rather speculatively, since a major contributor to odor unpleasantness is trigeminal stimulation, and garlic and fish are arguably the most pungent stimuli in the set, it might be suggested as a mediating factor. However, at odds with this suggestion are the results for mint, which shows a relatively strong trigeminal component and yet was on average judged as rather pleasant. Thus, the validity of this suggestion should be addressed in future studies.

Another possibility is that the correct identification of fish and garlic is facilitated by pungency or odor intensity, as such distinctly perceptible odors may be less prone to confusion than others. However, all of the 4 unpleasant odors were identified at similar rates (see **Figure 1**) even though there is wide variation in their mean perceived intensity (see Konstantinidis et al., 2006). Furthermore, although garlic and fish are rated as relatively more intense than coffee and clove, there is no obvious relationship between intensity and identification across the 16 odorants used in the Sniffin’ Sticks test (Konstantinidis et al., 2006). Hence, it seems relatively unlikely that intensity or pungency could have produced the observed pattern of results, compared with our suggested alternative regarding learning and familiarity.

### Correct Identification Percentages for Individual Odors

In line with previous studies (e.g., Boesveldt et al., 2008; Haehner et al., 2009), significant differences were noted for the individual odors in the percentages of correct identifications (see **Figure 1**). There is ample evidence that across the population of European adults, the Sniffin’ Sticks’ odor of turpentine, along with apple, lemon, and sometimes anise, quite invariably tend to be misidentified (Eibenstein et al., 2005; Konstantinidis et al., 2008; Haehner et al., 2009; Catana et al., 2012; Orhan et al., 2012). The poor performance on some odors might be due to their less prevalent real-life significance or, possibly, less realistic sensory representation in the Sniffin’ Sticks test. This could, at least, have been the case with apple, which was correctly identified by as few as one tenth of the participants. Another source of variation in cued odor identification tests is the nature of the distractor verbal labels provided. In some odors they might be more semantically or perceptually related to the target label than in other odors, which may, in turn, affect identification rates. Also, the unequal familiarity of the distractor verbal labels might have an impact on identification success rate as participants may use an exclusion heuristic to reach a correct answer without actually knowing the correct label. Although the Sniffin’ Sticks test is a widely used instrument both in research and clinical settings, to our knowledge the equality of the distractor labels has not been systematically assessed.

The issue of age-appropriateness of the items employed is specifically relevant to the present study. The Sniffin’ Sticks odor identification test has been successfully used with children before, including children as young as 3 years of age, with a success rate of 81% in children aged 6 years and over (Hummel et al., 2007a). In the olfactory tests deemed suitable for children, turpentine, and anise are not typically included but the other items have been successfully used in previous studies employing various other olfactory tests, both orthonasal and retronasal, with children as young as four-year-old (Richman et al., 1995; Monnery-Patris et al., 2009; Renner et al., 2009).

The effect of age on identification scores in our study was limited to only two odors (orange and apple). Taken at face value, this might be surprising as the effect of age is commonly reported in studies on odor identification in children (Richman et al., 1995; Ferdenzi et al., 2008; Monnery-Patris et al., 2009). The mostly negative findings reported here might be a consequence of the limited age range in our sample (8–11, with only three children being 11 years old). Further, in case of orange, which was the first item presented, the age effect might reflect a lack of concentration in the younger children at the beginning of the session.

Sex differences in odor identification, with women on average showing higher scores, have been repeatedly reported in adults (for reviews see Brand and Millot, 2001; Doty and Cameron, 2009) and some studies also found a similar pattern in prepubertal children (Richman et al., 1995; Ferdenzi et al., 2008; Monnery-Patris et al., 2009). Based on the current data, we found no significant differences in the overall identification score (data not shown, for details see Saxton et al., 2014). The negative results in our sample might be due to a limited statistical power as mean values were similar to those obtained by Ferdenzi et al. (2008) in French and Finnish children. When individual odors were analyzed separately, significantly higher scores in girls were found for the odor of orange. As identification scores in other odors showed no sex differences and the effect size in the case of orange was rather limited, we note that these results should be interpreted rather cautiously.

### Identification as a Proxy for Prior Experience

In the present study, odor identification was employed as a proxy for prior experience in order to overcome developmental differences in children’s use of various rating scales. In particular, younger children are more likely to respond at the extremes of rating scales (Chambers and Johnston, 2002) and, further, Berman et al. (1989) have suggested that even 8 to 10-year-olds tend not to assign ratings across the full range of the five-point rating scale. One might argue that for the sake of comparison, we could have collected both data on identification and familiarity ratings. However, we felt this was not achievable without compromising the quality of the collected data as attentional/perceptual capacity of the tested children is relatively limited.

However, the present approach also poses various methodological challenges. Most importantly, it is critical to consider the effect of the context provided by the odor label on olfactory perception and any subsequent ratings. Verbal labeling is known to modulate the perceived pleasantness of a given odor in adults and children alike (Bensafi et al., 2007), regardless of whether the identification has been correct or not (Ayabe-Kanamura et al., 1997), and whether or not the odor itself is actually presented (Herz, 2003). Therefore, in terms of the order of the tasks, we followed the procedure employed in previous studies (e.g., Distel et al., 1999; Degel et al., 2001; Sulmont et al., 2002) and obtained hedonic ratings first, before investigating what the participants knew about an odor’s identity. Nevertheless, a covert, unprompted identification attempt may have occurred during ratings of pleasantness, well before the participants were instructed to do so. Besides this, participants might hold multiple hypotheses about this identity (Cain et al., 1998) and if this were the case, it would be impossible to know which actually affected the pleasantness ratings.

Finally, in the present study, odor identification performance was, on a given trial, only coded as a “success” (1) or “failure” (0). Although some responses classified as “incorrect” might have been less of a miss than others, to be able to decide about the so-called near- and far-misses (Cain, 1979) one would have needed to know, among other things, the level of semantic similarity between the labels, as assessed specifically by this age cohort. Therefore, we caution that the reported correct identification percentages for the individual odors are not to be considered entirely synonymous with odor knowledge due to prior experience.

## Conclusion

The present study aimed to explore whether the previously reported positive relationship between odor pleasantness and olfactory knowledge can be generalized to an age group that clearly exhibits ongoing olfactory learning, using a cued odor identification task as a proxy for prior experience with odors. We found a positive effect for two of the unpleasant odors, but not for any pleasant ones. In order to be able to make robust generalizations about the relationship between odor pleasantness and knowledge in children, future studies should employ a wider range of odors with contrasting pleasantness, and labels for which a degree of semantic similarity can be inferred, and should assess familiarity and intensity of the tested odors.

## Conflict of Interest Statement

The authors declare that the research was conducted in the absence of any commercial or financial relationships that could be construed as a potential conflict of interest.
